# Investigation of Dracunculiasis Transmission among Humans, Chad, 2013–2017

**DOI:** 10.4269/ajtmh.20-0584

**Published:** 2020-12-07

**Authors:** Eugene W. Liu, Anita D. Sircar, Kolio Matchanga, Ada Mbang Mahamat, Neloumta Ngarhor, Philip Tchindebet Ouakou, Hubert Zirimwabagabo, Ernesto Ruiz-Tiben, Dieudonné Sankara, Ryan Wiegand, Sharon L. Roy

**Affiliations:** 1 Division of Parasitic Diseases and Malaria, Center for Global Health, Centers for Disease Control and Prevention, Atlanta, Georgia;; 2 Chad Office, World Health Organization (WHO), N’Djamena, Chad;; 3 Ministry of Public Health, N’Djamena, Chad;; 4 Guinea Worm Eradication Program, The Carter Center Atlanta, Georgia;; 5 Department of Control of Neglected Tropical Diseases, World Health Organization (WHO), Geneva, Switzerland

## Abstract

Dracunculiasis, slated for global eradication, typically is acquired by drinking stagnant water containing microscopic crustaceans (copepods) infected with *Dracunculus medinensis* larvae, causing clusters of case persons with worms emerging from the skin. Following a 10-year absence of reported cases, 9–26 sporadic human cases with few epidemiologic links have been reported annually in Chad since 2010; dog infections have also been reported since 2012. We conducted an investigation of human cases in Chad to identify risk factors. We conducted a case–control study using a standardized questionnaire to assess water and aquatic animal consumption, and links to dog infections. Case persons had laboratory-confirmed *D. medinensis* during 2013–2017. Each case person was matched to one to three controls without history of disease by age, gender, and residency in the village where the case person was likely infected. We estimated odds ratios (ORs) using simple conditional logistic regression. We enrolled 25 case persons with 63 matched controls. Dracunculiasis was associated with consumption of untreated water from hand-dug wells (OR: 13.4; 95% CI: 1.7–108.6), but neither with consumption of aquatic animals nor presence of infected dogs in villages. Unsafe water consumption remains associated with dracunculiasis. Education of populations about consuming safe water and using copepod filters to strain unsafe water should continue and expand, as should efforts to develop and maintain safe drinking water sources. Nevertheless, the peculiar epidemiology in Chad remains incompletely explained. Future studies of dogs might identify other risk factors.

## INTRODUCTION

Dracunculiasis (Guinea worm disease) is the first parasitic disease slated by the World Health Assembly for global eradication.^1^ It is typically spread by drinking stagnant water containing copepods (minute freshwater crustaceans) infected with the third-stage larvae of the roundworm *Dracunculus medinensis*. After 10–14 months of incubation, the adult female worm creates a painful blister on the skin. The blister bursts, exposing the anterior end of the worm. An infected host often immerses the lesion in water to relieve symptoms or does so in the course of daily activities, which induces the worm to expel larvae into the water. These larvae are in turn consumed by copepods, continuing the life cycle.^2^ Historically, the Guinea Worm Eradication Program’s interventions have addressed multiple points in the *D. medinensis* life cycle to prevent disease transmission: expanding access to safe drinking water free from infected copepods, distributing copepod filters to strain copepods from unsafe water sources, treating unsafe water sources with the chemical larvicide temephos to kill copepods, and detecting (through surveillance) and containing^3^ cases to prevent water contamination.

Chad is one of the few remaining countries in Africa where dracunculiasis is endemic. In 2000, the Chad Guinea Worm Eradication Program (CGWEP) reported no cases of dracunculiasis, prompting a change from active to passive surveillance. Although the change in surveillance could have accounted for the absence of cases in the ensuing decade, transmission was believed to have been interrupted.^4^ However, since 2010 when active surveillance was reinstated, 9–26 sporadic dracunculiasis cases in humans have been reported annually in Chad,^3,5–7^ with a low-level pattern of transmission and few epidemiologic links between cases.^8^ This contrasts with the typical transmission pattern where multiple cases are clustered in a community using a contaminated drinking water source. This sporadic pattern is of concern to the CGWEP because most cases are linked either to one another or to a particular water source, making it difficult to anticipate where new cases might occur, and reducing the likelihood that cases can be contained.

The sporadic pattern of transmission in Chad has been complicated by the appearance of an unprecedented number of dog infections. The annual count in dog infections has increased from 27 in 2012,^5^ when a village-based surveillance system was launched, to 1,935 dog cases in 2019.^7^ Worms isolated from these dogs have been found to be genetically indistinguishable from those isolated from humans,^9^ suggesting humans and dogs might share a common transmission pathway. Transport hosts, such as small copepod-feeding fish or amphibians, which harbor infective larvae that do not mature until they are ingested by a definitive host, or paratenic hosts that may consume infected copepods that infect host tissue but do not develop further, are proposed mechanisms to explain this peculiar pattern of transmission in Chad.^9,10^ Both dogs and humans (possibly less frequently) may be consuming raw or undercooked fish, their entrails, or other infected aquatic animals harboring *D. medinensis* larvae. Supporting this possibility is the observation that the highest incidence of dog infections overlaps with mass fish harvesting in lagoons and ponds that form along the Chari River, a major water source in Chad, at the end of the dry season.^9,10^ Dogs may have greater access to infected fish or fish entrails during this time. Further evidence supporting transport and/or paratenic host mechanisms includes experimental infection of tadpoles of the green frog *Lithobates clamitans*,^11^ isolation of *D. medinensis* larvae in four wild-caught frogs,^12^ and experimental infection of ferrets using fish transporting infected copepods in their intestines.^13^

Despite this hypothesized alternative mechanism of infection, a 2012 investigation in Chad did not identify an association between human dracunculiasis and aquatic animal consumption.^14^ However, the 2012 investigation did find that drinking water from secondary sources was associated with transmission of *D. medinensis*. Secondary water sources were those used on a regular basis in addition to the single main water source used at home on a daily basis. These secondary drinking water sources typically were used outside the village of residence, for example, during farming or fishing. However, these water-related findings do not fully explain the unusual epidemiologic patterns in humans in Chad.

The occurrence of ongoing cases and unclear mechanisms of *D. medinensis* transmission in humans is a challenge to eradication efforts in Chad and to global eradication. An understanding of the risk factors for transmission is urgently needed, particularly those factors that might be shared between humans and dogs. In an attempt to identify these factors, we conducted an investigation of *D. medinensis* transmission in humans in Chad.

## METHODS

### Study design and participants.

We conducted a matched case–control study to assess water and aquatic animal consumption. Case persons were individuals with a worm extracted during 2013 to mid-2017, identified as Guinea worm by a CGWEP supervisory staff member, and with confirmatory laboratory diagnosis as *D. medinensis* at the U.S. CDC.

Each case person was matched to one to three controls by age-group (0–5 years, 6–14 years, 15–25 years, 26–35 years, 36–49 years, and 50 years and older), gender, and shared residency in the likely village of transmission during the period of infection (POI). We defined the POI as the 4-month period occurring from 10 to 14 months before emergence of the first worm. We defined the possible village of transmission as the self-identified single location/village where the case person spent the most time during the POI. Within each village, we selected controls based on the nearest household to that case person. We excluded potential controls if they or household members, including domestic animals, ever had a Guinea worm emerge based on self-report. We selected only one control per household. If multiple persons in the household met the selection criteria, we selected the person closest in age to the case person and of the same gender. If a potential control was not at home nor available, we continued with household selection using expanding concentric circles of distance from the case person’s household. We repeated this process until one to three consenting eligible controls were successfully enrolled and interviewed, or logistical constraints prevented further enrollment.

### Questionnaire.

A standardized questionnaire addressing activities and practices performed during the POI, focusing on drinking water sources, consumption of aquatic animals, and traditional practices involving aquatic animals, was used to interview both case persons and controls. We also asked whether the respondents had ever seen dogs with emerging Guinea worms in their households or communities. We categorized drinking water sources into those safe and unsafe from possible contamination with copepods infected with *Dracunculus* larvae. Safe water sources were defined as taps, boreholes with pumps, rivers/streams (which because of their flowing nature prevent copepods harboring L1 larvae from developing into infective L3 larvae), springs protected by walls, rainwater catchments, bottled or bagged water, and water cisterns. Unsafe water sources were stagnant water sources where copepods might live and included lakes/dams/swamps, ponds/pools, hand-dug wells, unprotected springs, canals, and brick ponds (i.e., ponds from which mud was removed to make bricks). Use of an unsafe water source prompted follow-up questions about water treatment (e.g., boiling, filtering, and/or chlorination) before consumption. We also asked what unsafe water sources outside the home may have been used when working in the fields, while traveling, or at school. Finally, because of the long period of recall inherent to dracunculiasis due to its 10–14 month incubation period, the previous questions referenced activities and practices generally performed during the 4 months of the year that the case person’s POI occurred. The questionnaire was administered verbally in French or interpreted into the participant’s local language. To assist with interpretation and recall, we used job aids consisting of laminated cards with photographs of the various types of aquatic animals (including fish, waterfowl, crustaceans, reptiles, and other animals) and water sources during the interviews. Questionnaire responses were recorded on mobile devices.

### Ethical approval.

This investigation (Protocol #2017-250) was approved as a non-research public health emergency response by the Office of the Associated Director for Science, Center for Global Health at the CDC. The investigation was conducted in accordance with the Declaration of Helsinki and complied with U.S. government regulations for protecting patient privacy. The Minister of Public Health in Chad granted permission for this investigation.

We obtained informed written consent from all participants before administration of the questionnaire. For children younger than 18 years, we obtained permission to participate in the investigation from a parent or guardian; assent was obtained from children aged 7–17 years. A parent or guardian of children younger than 15 years was present during the interview to assist with answering questions.

### Statistical analysis.

Analysis was performed with *R* statistical software^15^ and the *survival* package.^16,17^ Demographic differences between eligible case persons who were enrolled and not enrolled in the study were evaluated using Fisher’s exact test. Of those enrolled, differences in proportions were also evaluated using Fisher’s exact test for unmatched demographics. We performed conditional logistic regression by single factors to calculate odds ratios and identify potential risk and protective factors for dracunculiasis.

## RESULTS

### Study participant demographics.

**Figure 1.**
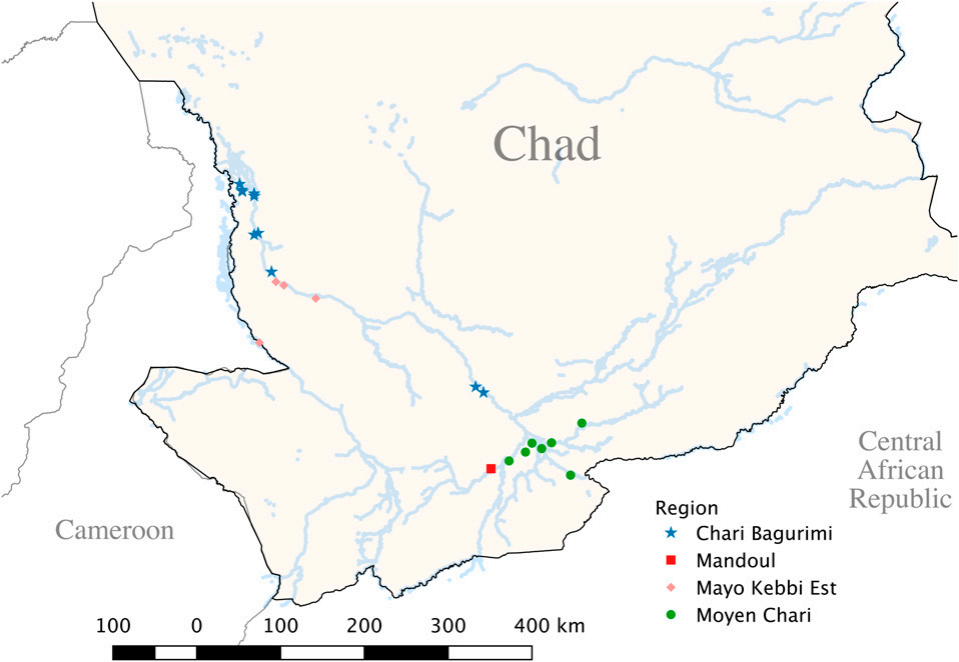
Map of the southwest region of Chad showing 25 villages* from four regions along the Chari River system that were visited in July 2017 for a matched case–control study of 25 case persons with patent dracunculiasis infections from 2013 to 2017 and 63 controls. *In this map, three pairs of villages are adjacent to each other and share the same GPS coordinate. This figure appears in color at www.ajtmh.org.

The CGWEP identified 48 case patients in the 4 years before July 2017 when this study was conducted. Of these, we interviewed 23 (48%), as well as two others from early 2013 and late 2017, who were all we could reach given logistical and security constraints. We interviewed 63 controls matched to case persons by village of infection, gender, and age-group. All interviews occurred in 25 villages in four different regions (Figure 1). The village of detection and village of exposure/infection were discordant for nine case persons because of migration of these individuals during the 10- to 14-month incubation period. Overall, 52% of case persons and 48% of controls were female; the plurality of case persons (44%) and controls (46%) were in the 6- to 14-year age-group. The plurality of case persons self-identified to the Sara Madjigay ethnicity (24%), whereas the plurality of controls self-identified to the Sara ethnic group (33%). A plurality of case persons were farmers (48%), whereas a plurality of controls were pupils (excluding high school and college students) (36%) (Table 1). There were no significant differences in proportions by ethnicity or occupation by Fisher’s exact test (*P* < 0.1 and *P* < 0.4, respectively).

**Table 1:** Demographic characteristics of 25 case persons and 63 matched controls in a study of human dracunculiasis—Chad, 2013–2017

Factor	% Of cases	*n* of 25	% Of controls	*n* of 63
Gender				
Female	52	13	48	30
Male	48	12	52	33
Age-group (years)				
0–5	4	1	5	3
6–14	44	11	46	29
15–25	24	6	25	16
26–35	4	1	3	2
36–49	8	2	6	4
≥ 50	16	4	14	9
Ethnicity				
Sara group*	64	16	57	36
Massa and Mousgoum	12	3	8	5
Arabe	0	0	8	5
Other†	24	6	27	17
Occupation				
Farmer	48	12	29	18
Pupil	24	6	36	23
Child (not a pupil)	8	2	10	6
Fisher	8	2	8	5
Merchant/business owner	4	1	2	1
Elderly/handicapped/with chronic illness	4	1	0	0
Student in high school or college	0	0	10	6
Herdsman	0	0	2	1
Other	4	1	5	3

No significant differences by Fisher’s exact test in unmatched demographic characteristics (ethnicity and occupation) between case persons and controls were found.

* The Sara group includes Sara, Sara Kaba, Sara Madjigay, Mbaye, Ngambaye, Mberi, Goulaye, Mongo, and Laka.^18^

† Other ethnicities consist of Baguirmi, Boa, Boulala, Briguite (Abdeya), Mboulou, and Ngor.

### Nonrespondents.

Of the 25 cases persons who could not be interviewed, nine were in the Salamat region to which we could not travel because of security restrictions; 14 were identified by the CGWEP as living in areas too remote, inaccessible, or insecure; and two were inaccessible in the field. As expected, enrolled case persons differed from non-enrolled case persons by regions, districts, and villages of residence and exposure and by geography-associated ethnicity. However, there were no statistically significant differences between enrolled and non-enrolled case persons with respect to age, gender, number of emerging worms, or occupation (Supplemental Table 1).

### Association between water sources and dracunculiasis.

We found that 92% of case persons versus 95% of controls consumed water from safe sources during the POI (Table 2). No safe water source was associated with dracunculiasis on simple conditional logistic regression. By contrast, 96% of case persons versus 78% of controls consumed water from unsafe sources during the POI. No case persons treated unsafe water, whereas three controls treated unsafe water with cloth filters; thus, 73% of controls consumed untreated and unsafe water. Water consumption from untreated hand-dug wells was associated with dracunculiasis on simple conditional logistic regression (OR: 13.4; 95% CI: 1.7–108.6). Similarly, with respect to water from unsafe sources (e.g., hand-dug wells, unprotected springs, lakes/dams/swamps, ponds/pools, canals, or brick ponds) consumed outside the home, only consumption of water from hand-dug wells was associated with cases (OR: 5.7; 95% CI: 1.2–27.8). No sources of unsafe water consumed inside the home were associated with dracunculiasis.

**Table 2:** Associations between consumption of water from different sources and development of dracunculiasis in humans, estimated with simple logistic regression models in a matched case–control study—Chad, 2013–2017

Factor	% Of cases	*n* of 25	% Of controls	*n* of 63	odds ratio
Safe water consumption	92	23	95	60	0.8 (0.1–6.4)
Tap	12	3	10	6	1.7 (0.3–9.3)
Borehole	48	12	52	33	1.1 (0.2–5.8)
Protected spring	0	0	2	1	0.0 (0.0–∞)
Rainwater	24	6	44	28	0.3 (0.1–1.1)
Bottled/bagged water	28	7	49	31	0.3 (0.1–1.2)
Water cistern	12	3	21	13	0.5 (0.1–2.0)
River/stream	84	21	68	43	2.6 (0.8–8.9)
Unsafe and untreated water consumption	96	24	73	46	9.0 (1.1–72.9)*
Hand-dug well	80	20	51	32	13.4 (1.7–108.6)*
Unprotected spring	8	2	17	11	0.4 (0.1–1.9)
Lake/dam/swamp	28	7	35	22	0.6 (0.2–1.9)
Pond/pool	60	15	51	32	1.5 (0.5–4.5)
Canal	24	6	14	9	1.9 (0.5–6.4)
Brick pond	12	3	6	4	6.0 (0.5–66.2)
Unsafe water consumed inside the home	48	12	52	33	0.5 (0.1–2.0)
Hand-dug well	40	10	40	25	0.7 (0.1–3.0)
Unprotected spring	0	0	2	1	0.0 (0.0–∞)
Lake/dam/swamp	0	0	11	7	0.0 (0.0–∞)
Pond/pool	8	2	10	6	0.8 (0.1–7.8)
Canal	4	1	2	1	3.0 (0.2–48.0)
Brick pond	4	1	3	2	3.9E7 (0.0–∞)
Unsafe water consumed outside the home	92	23	76	48	3.8 (0.8–19.2)
Hand-dug well	72	18	49	31	5.7 (1.2–27.8)*
Unprotected spring	8	2	16	10	0.5 (0.1–2.3)
Lake/dam/swamp	28	7	38	24	0.5 (0.2–1.6)
Pond/pool	60	15	52	33	1.4 (0.4–4.4)
Canal	24	6	17	11	1.4 (0.5–4.4)
Brick pond	12	3	6	4	6.0 (0.5–66.2)

* *P* < 0.05.

We also asked individuals what single water source they used most often during the POI. The plurality of both case persons and controls reported using hand-dug wells most often, followed by boreholes (Table 3). There was no significant difference in proportions between case persons and controls for the most commonly used water sources by Fisher’s exact test. Similarly, on simple logistic regression, there was no association between the type of water source used most often and the development of dracunculiasis.

**Table 3:** Associations between different water sources used *most often* and development of dracunculiasis estimated in two simple logistic regression models, the first with the water source used most often as a factor and the second with these sources grouped into safe and unsafe—Chad, 2013–2017

Water source used most often	% Of cases	*n* of 25	% Of controls	*n* of 63	odds ratio
Borehole*	28	7	32	20	NA (reference level)
Tap*	0	0	6	4	0.0 (0.0–∞)
Rainwater*	0	0	2	1	0.0 (0.0–∞)
River/stream*	16	4	13	8	∞ (0.0–∞)
Hand-dug well†	44	11	40	25	1.2 (0.1–26.0)
Pond/pool†	12	3	8	5	∞ (0.0–∞)
Safe	44	11	52	33	NA (reference level)
Unsafe	56	14	48	30	1.8 (0.4–9.1)

* In the second logistic regression model, this water source was considered safe.

† In the second logistic regression model, this water source was considered unsafe.

### Association between consumption of aquatic animals and dracunculiasis.

Nearly all case persons and controls (except for one control) reported consuming fish (Table 4), including common varieties (carp, sardine, and sole) and preparations (salanga—small fish that are sun-dried) found in Chad, limiting the possibility of identifying associations between fish consumption and dracunculiasis. A minority of both case persons and controls reported consuming fish entrails; we found no association between entrail consumption and dracunculiasis. We found no association between dracunculiasis and consumption of small lizards, monitors, skinks, frogs (including three common varieties referred to as black, yellow, and green), turtles/tortoises, snakes, crustaceans, or waterfowl. We found no associations between dracunculiasis and consumption of different preparations of aquatic animals (cooked, dried, smoked, grilled, undercooked, or raw).

**Table 4:** Associations between consumption of aquatic animals and development of dracunculiasis in humans estimated with simple logistic regression models in a matched case–control study—Chad, 2013–2017

Factor	% Cases	*n* of 25	% Controls	*n* of 63	odds ratio
Fish	100	25	98	62	∞
Entrails	36	9	35	22*	1.0 (0.3–2.6)
Cooked	88	22	87	55	NA
Dried	76	19	46	29	∞ (0.0–∞)
Smoked	80	20	51	32	∞ (0.0–∞)
Grilled	76	19	71	45	2.4 (0.4–14.0)
Undercooked	40	10	27	17	2.1 (0.6–6.9)
Raw	0	0	2	1	0.0 (0.0–∞)
Lizards	4	1	0	0	∞ (0.0–∞)
Water monitors	88	22	75	47	2.4 (0.6–9.6)
Skinks	4	1	0	0	∞ (0.0–∞)
Frogs	24	6	16	10	1.8 (0.5–6.5)
Turtles	52	13	49	31	1.0 (0.4–3.0)
Snakes	16	4	5	3	7.8 (0.8–74.0)
Crustaceans	0	0	2	1	0.0 (0.0–∞)
Waterfowl	80	20	75	47	1.3 (0.3–5.3)

* Out of 62 (rather than 63).

### Association between dog ownership and dracunculiasis.

Given the occurrence of dracunculiasis infections in dogs, we also asked about dog ownership. Only one case person reported having a dog with dracunculiasis (ownership of a dog with dracunculiasis was an exclusion criterion for controls). There was no association between seeing a dog with dracunculiasis in the community and having dracunculiasis oneself (OR: 1.8; 95% CI: 0.6–5.9).

## DISCUSSION

In this matched case–control study, we attempted to identify risk factors for human dracunculiasis in Chad. In addition to asking about consumption of drinking water, a known transmission pathway for *D. medinensis*, we asked about preparation and consumption of food to examine the possibility of a novel transmission pathway associated with paratenic or transport hosts that could also explain infections in dogs that are genetically indistinguishable with those in humans. Although we found no clear association between dracunculiasis and consumption of aquatic animals, or seeing infected dogs in the community, we cannot rule out the possibility of a paratenic or transport host to explain the sporadic infection in humans, given our small sample size. From participant responses to an a priori list of drinking water sources, we identified associations between consumption of untreated water from hand-dug wells with dracunculiasis in humans. However, for the single water source used most often, no specific water sources were associated with dracunculiasis, including hand-dug wells that were used by a plurality of case persons and controls as their main sources of water.

Intermittent consumption of hand-dug well water containing *D. medinensis*–infected copepods, consumed while away from home, could possibly explain some of the current epidemiology in Chad where human cases generally occur in isolation. This unusual pattern might reflect the location of consumption of contaminated water in isolated areas on farms or in sites distant from villages and other domestic or commercial settings (e.g., along paths taken for hunting or travel to other locations). Water consumption from such isolated wells would be intermittent, and consequently, few persons would be exposed and infected by contaminated water. This is in contrast to a large geographic cluster of cases associated with a single village that would occur when a village’s common water supply becomes contaminated. Intermittent or single consumption events could explain the pattern of one or a few isolated cases in multiple locations without easily discernible epidemiologic links between them. The association that we found between dracunculiasis and intermittent water consumption from hand-dug wells while outside the home is also consistent with a finding from a case–control study in Chad in 2012.^13^ In this prior study, dracunculiasis was significantly associated with water consumption from secondary water sources (defined as regularly used water sources in addition to the single main daily water source used at home). Within the category of secondary water sources, use of water from hand-dug wells was associated with dracunculiasis, as was water from lagoons or ponds.

Our water-associated findings must be interpreted with caution. The water sources we referred to in our questionnaire may have been subject to different interpretations by respondents. Respondents who indicated that they had consumed water from the Chari River could have consumed water containing infected copepods from a stagnant part of the river isolated from the main river, which may harbor copepods and be an unsafe source of water. We did not differentiate between hand-dug wells with and without sealed walls around them to prevent inflow of surface water. Furthermore, because of our small sample size, we did not account for multiple comparisons in our analyses that could have resulted in false associations. Nevertheless, the association between dracunculiasis and hand-dug wells remains, despite inclusion of wells surrounded by protective walls that would not be at risk for surface water contamination during flooding. Testing of hand-dug wells for copepods could help determine whether the observed association with dracunculiasis may be due to well contamination with these organisms or to confounding with another variable associated with the use of hand-dug wells.

This study had other limitations. Given the limited numbers of total case persons and the security and logistical constraints, we were able to include only a limited number of cases. This limited our ability to identify important associations and prevented us from performing multivariable modeling. Another limitation was the need for participants to describe risks occurring up to 14 months before worm emergence in case patients, which may have been up to 4 years before the interview. This may have led to recall bias among participants, with participants providing responses relating to seasonal habits, rather than activities performed during the specific time of inquiry. We are not aware of any disruptions to well-defined seasonal patterns of village life that would prevent these responses of habitude from describing activities particular to the year of infection. Furthermore, although the possible village of transmission was self-identified by the case person as the single village/location where he/she spent the most time during the POI, it may not have been the actual location of transmission. Finally, we defined controls as individuals who never had worms emerge from their skin. Thus, there is a possibility that they may have had sub-patent infections without worm emergence. The presence of controls with non-patent infections, which would result in overmatching, could thus limit our ability to detect associations between dracunculiasis and risk factors in our study.

Our finding of an association between dracunculiasis and water consumption from an unsafe source supports the utility of the CGWEP continuing to support development and repair of safe drinking water sources and to train and educate populations about consuming drinking water from safe sources. The distribution of fine mesh cloth filters to strain out infected copepods from contaminated drinking water, as well as the distribution of pipe filters for those consuming water outside the home, would likely be of benefit in villages with dracunculiasis cases in humans or animals with evidence of recent or ongoing transmission. Although prevailing evidence from two case–control studies continues to point to water as an important vehicle for dracunculiasis transmission in humans in Chad, the peculiar epidemiology in which cases are not clustered around a specific contaminated water source remains unexplained. Future studies of dogs and their owners may assist in identifying shared risk factors between the two hosts.

## Supplemental table

Supplemental materials
