# Supplementary material for: Investigation of Dracunculiasis Transmission among Humans, Chad, 2013–2017
Source: Am J Trop Med Hyg. 2020 Dec 7;104(2):724–30. doi: 10.4269/ajtmh.20-0584 (PMC7866328; doi:10.4269/ajtmh.20-0584)
Supplement: Supplementary file 1 [file tpmd200584.SD1.docx]

**Supplemental Table 1:** Comparison of demographic characteristics between 25 enrolled and 25 non-enrolled case-persons in a study of human dracunculiasis—Chad, 2013–2017*

| **Factor** | **% of enrolled** | **(n of 25)** | **% of non-enrolled** | **(n of 25)** | **P-value** ^†^ |
| --- | --- | --- | --- | --- | --- |
| Sex |  |  |  |  | 1 |
| Female | 52 | (13) | 56 | (14) |  |
| Male | 48 | (12) | 44 | (11) |  |
|  |  |  |  |  |  |
| Age group (*in years*) |  |  |  |  | 0.87 |
| 0–5 | 8 | (2) | 12 | (3) |  |
| 6–14 | 52 | (13) | 36 | (9) |  |
| 15–25 | 12 | (3) | 20 | (5) |  |
| 26–35 | 4 | (1) | 8 | (2) |  |
| 36–49 | 8 | (2) | 12 | (3) |  |
| >50 | 16 | (4) | 12 | (3) |  |
|  |  |  |  |  |  |
| Ethnicity |  |  |  |  | <0.00073 |
| Sara group^§^ | 76 | (19) | 32 | (8) |  |
| Massa and Mousgoum | 12 | (3) | 8 | (2) |  |
| Arabe | 0 | (0) | 12 | (3) |  |
| Kabalaye | 0 | (0) | 4 | (1) |  |
| Kibet, Rachid, and Rounga | 0 | (0) | 32 | (8) |  |
| Other** | 12 | (3) | 12 | (3) |  |
|  |  |  |  |  |  |
| Occupation |  |  |  |  | 0.37 |
| Child | 60 | (15) | 36 | (9) |  |
| Farmer | 12 | (3) | 8 | (2) |  |
| Fisher | 4 | (1) | 8 | (2) |  |
| Housewife | 12 | (3) | 36 | (9) |  |
| Multiple ^††^ | 4 | (1) | 4 | (1) |  |
| Other^§§^ | 8 | (2) | 8 | (2) |  |
|  |  |  |  |  |  |
| Number of worms |  |  |  |  | 0.80 |
| 1 | 76 | (19) | 72 | (18) |  |
| 2 | 8 | (2) | 16 | (4) |  |
| 3 | 16 | (4) | 12 | (3) |  |
|  |  |  |  |  |  |
|  |  |  |  |  |  |

* CGWEP identified 48 case-patients in the 4 years prior to July 2017 when this study was conducted. Of the 48 case-persons identified during mid-2013–mid-2017, we interviewed 23 (48%), who were all we could reach given logistical and security constraints. The two additional case-persons were interviewed who were not part of this cohort of 48 but were identified in early 2013 and late 2017. Therefore, a total of 25 case-persons were enrolled. Of the 25 cases-persons who could not be interviewed, 9 were in the Salamat region to which we could not travel due to security restrictions, 14 were identified by CGWEP as living in areas too remote, inaccessible, or insecure, and 2 were inaccessible in the field. These 25 case-persons were non-enrolled. Of note, there were a total of 67 cases in 2013-2017 strictly by year. However, our study accounted for 48 cases in the 4 years prior to July 2017 when our study was conducted (mid-2013–mid-2017), as well as two additional case-persons identified in early 2013 and late 2017, respectively. Thus, 17 cases in 2013 and 2017 were not considered for enrollment in our study as they were outside the mid-2013–mid-2017 timeframe. Data for the analyses in this table were taken from the Chad Guinea Worm Eradication Program (CGWEP) surveillance system database, which is the only source of information available for the non-enrolled case-persons. Therefore, some of the age, ethnicity, and occupation categories are different from those used in our study. Additionally, some case-patients in our study may have provided different demographic information to our interviewers (e.g., age, ethnicity, occupation) than to the CGWEP surveillance system so there may be some discrepancies between data in this supplementary table and data in Table 1, representing differences between the two data sources.

^†^ Variables were evaluated using Fisher’s exact test because of the small sample size and cell sizes <5.

^§^ The Sara group includes Sara, Sara Kaba, Sara Madjigay, Mbaye, Ngambaye, Mberi, Goulaye, Mongo, and Laka^18^

** Other ethnicities consist of Baguirmi, Boa, Boulala, Briguite [Abdeya], Mboulou, and Ngor

^††^ Multiple included one enrolled case-person who was a farmer/fisher and one non-enrolled case-person who was a farmer/fisher/housewife

^§§^ Other occupations included butcher and stock breeder for enrolled case-persons, and mason and potter for non-enrolled case-persons
